# The Contribution of Hepatic Macrophage Heterogeneity during Liver Regeneration after Partial Hepatectomy in Mice

**DOI:** 10.1155/2022/3353250

**Published:** 2022-10-07

**Authors:** Pengfei Ma, Wenchao Zhao, Chunchen Gao, Zhiwei Liang, Longshuan Zhao, Hongyan Qin, Xiaolin Sun

**Affiliations:** ^1^Department of Hepatopancreatobiliary Surgery, The First Affiliated Hospital of Zhengzhou University, Zhengzhou 450000, China; ^2^State Key Laboratory of Cancer Biology, Department of Medical Genetics and Developmental Biology, Fourth Military Medical University, Xi'an 710032, China; ^3^Faculty of Hepatopancreatobiliary Surgery, Chinese PLA General Hospital, Beijing 100080, China; ^4^Department of Hypertension, Henan Provincial People's Hospital, Zhengzhou 450003, China

## Abstract

**Methods:**

In the present study, we investigated hepatic macrophage heterogeneity in murine liver regeneration after 2/3 PHx through immunofluorescence staining, fluorescence-activated cell sorting analysis, and quantitative reverse transcription-polymerase chain reaction.

**Results:**

Our research showed that Kupffer cells reduced rapidly in the early PHx and restored gradually depending on local proliferation and replenishment from infiltrating monocyte-derived macrophages. The ratio of ly6C^hi^ to ly6C^lo^ subset of macrophages in the liver changed dynamically, and hepatic macrophage function exhibits a significant difference in different stages of liver regeneration. Moreover, blocking infiltrating monocyte-derived macrophage recruitment augmented Kupffer cell proliferation but impaired the restoration of the hepatic macrophage pool, which led to delayed hepatocyte mitosis and liver regeneration.

**Conclusions:**

Our data suggest that hepatic macrophage changes dynamically in origin and function during liver regeneration following PHx and macrophage-targeted liver regeneration should consider macrophage heterogeneity.

## 1. Introduction

The liver is the largest internal organ of the human body. It is not only a material metabolism center but also an important synthesis, secretion, and immune barrier organ. In addition, the liver has a remarkable ability to regenerate after tissue loss. Different from the traditional definition of “regeneration,” liver regeneration is not to rebuild the lost liver lobe, but mainly through the hypertrophy and proliferation of mature hepatocytes to achieve volume recovery. Liver regeneration is a sophisticated and well-coordinated pathophysiological procedure that allows the liver to regain its metabolic and synthesis activities in a specific and well-timed manner. After 2/3 partial hepatectomy (PHx) which is the most frequently used model of liver regeneration, hepatocytes are the first to proliferate and this process lasts 3 days with a peak at about the second day in mice. And then, the other hepatic cells enter into deoxyribonucleic acid (DNA) synthesis subsequently and reconstitute the regenerative liver mass. The whole process lasts about 8 days in mice, which is accompanied by significant and orderly changes of many cytokines, growth factors, and metabolic networks [1].

Hepatic macrophage is a heterogenetic population in origin and function which plays multiple and important roles in liver homeostasis and pathology [2]. According to their origin, liver macrophages can be divided into yolk sac-derived Kupffer cells (KCs) and infiltrating monocyte-derived macrophages (IMs). KCs, as liver-resident macrophages, exert integral features for the duration of homeostasis, including iron balance regulation, removal of cell debris and intestine-derived pathogens, and liver immune tolerance maintenance. KCs are primarily identified as CD45^+^F4/80^high(hi)^CD11b^+^ cells in the mouse, and a part of them are CD68 positive [3]. KCs dominate the hepatic macrophage pool at a steady state and constantly renew themselves, independently of bone marrow progenitors. In the healthy liver, only a few of hepatic macrophages descend from circulating monocytes. However, under inflammation state, a large number of IMs migrate into the liver and are recognized as CD45^+^F4/80^+^CD11b^hi^ cells [3]. More importantly, IMs harbor fascinating plasticity according to the tissue niche and microenvironment, particularly during complicated pathological processes. In different stages of liver diseases [4, 5], IMs render distinctly functional heterogeneity according to the expression level of ly6C. The ly6C^hi^ IMs have been proposed as potent proinflammatory cells, while ly6C^lo^ cells serve as restorative macrophages and participate in the regeneration of liver-resident macrophages [6]. In addition, some studies proposed that ly6C^hi^ IMs could represent precursors of ly6C^lo^ IMs [5].

Hepatic macrophages are known to participate in the coordination of all phases of liver regeneration after PHx [7]. In the inductive phase (0–3 days after PHx), macrophage activation is recommended and affords the preliminary priming pressure for hepatocyte proliferation. Macrophage-derived cytokines, tumor necrosis factor-*α* (TNF-*α*), and interleukin 6 (IL-6) are essential components in early liver regeneration, and hepatocyte DNA synthesis is suppressed in mice carrying antagonist or deletion of these genes [8]. During the subsequent angiogenic phase (4–8 days postoperation), macrophages participate in the regulation of vascular sprouting and formation of new vessels, which are related with the secretion of angiopoietin and vascular endothelial growth factor (VEGF) [9]. Besides, the selective depletion of hepatic macrophages at the different time points has the different influence on liver regeneration after PHx [10, 11]. These studies suggest that the complicated function heterogeneity of hepatic macrophages exist in liver regeneration after PHx. However, as yet, there is no study focused on the dynamics of different macrophage subsets, such as the different origin and function of macrophages, after PHx.

Therefore, the purpose was to explore the heterogeneity of liver macrophages while there was liver regeneration in mice following 2/3 PHx. Our research showed that KCs reduced rapidly in the early PHx and restored gradually depending on proliferation locally and replenishment from IMs. The ratio of ly6C^hi^ to ly6C^lo^ subset of IMs changed dynamically, and hepatic macrophage function was significantly different at different stages of liver regeneration. Moreover, blocking IM recruitment augmented KC proliferation but impaired the restoration of the hepatic macrophage pool, and ultimately, it delayed hepatocyte mitosis leading to the failure of liver regeneration.

## 2. Materials and Methods

### 2.1. Animal Models

C57BL/6 mice and C-C chemokine receptor 2 (CCR2) knockout (KO) mice (CCR2^−/−^, stock # 004999, Jackson Laboratory) were housed in a specific pathogen-free environment with a 12–12 light-dark cycle. 8–12-week-old male mice received 2/3 PHx. PHx was performed as described [12]. In brief, mice were anesthetized with 0.6% pentobarbitone sodium injected subcutaneously. After disinfection, mice underwent midline laparotomy. And then, the left and middle lobes of the liver were consecutively ligated at the base and resected. The abdominal wall and the skin were sutured separately. Sham-operated mice only underwent laparotomy followed by abdomen closure. Sham-operated mice were used as control (Ctrl), and data from them are included in graphs at the “D0” time point.

All animal experiments were approved by the Ethics Committee of Zhengzhou University and Fourth Military Medical University. All animal were used in accordance with the Guide for the Care and Use of Laboratory Animals prepared by the National Academy of Sciences and published by the National Institutes of Health (NIH publication 86-23, revised 1985).

### 2.2. Histology

Immunohistochemistry (IHC) and immunofluorescence (IF) staining were carried out in accordance to standard protocols as previously described [12]. All antibodies for staining have been listed in Supplementary Table 1. And the Hoechst 33258 (Sigma Chemical Co., St. Louis, MO, USA) was used to counterstain nuclei. Photographs were taken using a microscope (BX51, Olympus, Tokyo, Japan) with a CCD camera (DP70, Olympus).

### 2.3. Cells Isolation

Hepatic nonparenchymal cell (HNPCs) isolation was performed as previously described [2]. Mice were perfused with 20 mL phosphate-buffered saline (PBS) through the hepatic portal vein. A part of liver tissues (about 0.5 g) was harvested, minced, and incubated in a digestion buffer (Hank's balanced salt solution with calcium and magnesium (HBSS) plus 0.5 mg/mL collagenase IV (Sigma) and 100 *μ*g/mL DNase (Roche, Basel)) for 30 min at 37°C. Digested livers were disrupted on the 100-mesh metal strainer, and then, the cell suspensions were passed through a 200-mesh filter. Hepatic cells were collected by centrifugation at 300 × g for 5 min and then resuspended in RPMI 1640 with 2% FCS to inactivate the enzymes. To remove hepatocytes, three times centrifugation at 50 × g for 3 min were completed. The remaining cells were resuspended in 30% Percoll (Solarbio, Beijing), followed by centrifugation at 450 × g for 20 min to remove cell debris. After lysis of red blood cells, the remaining HNPCs were resuspended and counted for further analyses.

Bone marrow (BM) and peripheral blood (PB) leukocytes were isolated by routine method. In a nutshell, BM cells were evacuated from mice's femurs and tibias and mechanically disseminated. A 200-mesh filter was used to filter the cell suspensions. Red blood cells were lysed using the ACK lysis buffer (Comwin, Beijing), and other cells were centrifuged, which were considered as BM leukocytes. Peripheral blood was collected by eyeball extirpating. After lysis of red blood cells and centrifugation at 300 × g for 4 min repeatedly, the deposits were resuspended for further analyses.

### 2.4. Fluorescence-Activated Cell Sorting (FACS) Analysis

Cells were resuspended in FACS buffer and cultured with anti-rat Fc receptor (CD16/32) antibody (Abcam Technology, Cambridge, UK) for 10 min to minimize nonspecific antibody binding. Cells were then stained with primary antibodies and secondary antibodies (Supplementary Table 1) routinely. Dead cells were excluded by propidium iodide (PI) (BD Pharmingen). FACS analysis was performed using a FACSCalibur™ flow cytometer (BD Immunocytometry Systems). Data were analyzed with the FlowJo7.6.1 software (TreeStar, Ashland, OR).

### 2.5. Magnetic-Activated Cell Sorting (MACS)

MACS was performed by using the BD IMag™ cell separation system [12]. HNPCs treated with normal rat serum were incubated with biotinylated anti-mouse F4/80 antibody for 30 min at 4°C, followed by incubation with microbeads (Streptavidin Particles Plus-DM, BD) for 20 min at 4°C. The labeled cells were mixed in 1.5 mL IMag buffer, and then, the tubes were fixed in the BD IMagnet for 30 min at 4°C. The positive fraction cells adsorbed on the tube walls were collected, resuspended in 1.5 mL IMag buffer, and purified by IMagnet isolation again. The separated positive cells were collected for further analyses.

### 2.6. RNA Extraction and qRT-PCR

Total RNA was extracted using the TRIzol reagent (Invitrogen, Waltham, MA) in accordance to the instructions. For MACS isolated cells, RNA was prepared using the RNeasy MicroPlus Kit (QIAGEN Sciences, Germantown, MD). Reverse transcription PCR and qRT-PCR were sequentially performed. Genes were amplified with the SYBR Premix EX Taq™ II Kit (TaKaRa, Dalian, China), with *β*-actin as an internal control. The specific primers are shown in Supplementary Table 2 (Sangon Biotech, Shanghai, China).

### 2.7. Statistical Analysis

Images were treated using ImagePro Plus 5.1 software (Media Cybernetics Inc., Bethesda, MD). Data were analyzed with GraphPad Prism 5 software (San Diego, CA). Unpaired Student's *t*-test or paired *t*-test was carried out to comparing the differences among groups. It is markedly different when *P* < 0.05.

## 3. Results

### 3.1. Dynamic Change of the Hepatic Macrophage Number after PHx

**Figure 1:**
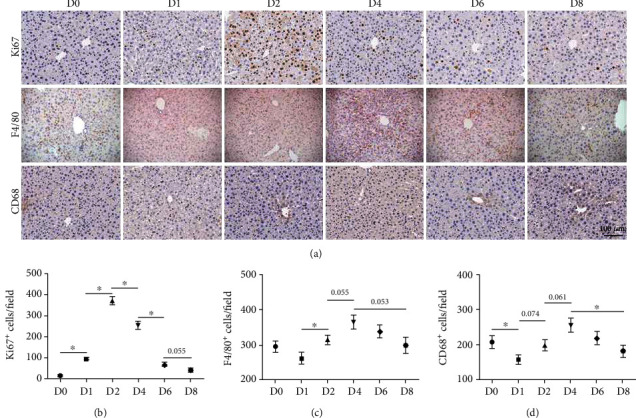
The dynamic change of the number of macrophage subsets after PHx. (a) C57BL/6 mice were sacrificed at day 1, 2, 4, 6, or 8 after 2/3 PHx or sacrificed immediately after sham operation. Liver sections were stained with anti-Ki67, anti-F4/80, or anti-CD68 IHC. The brown grains indicated the positively stained regions. The number of (b) Ki67^+^ cells, (c) F4/80^+^ cells, and (d) CD68^+^ cells in (a) were counted and quantitatively compared. Bars = means ± SD, *n* = 8. ^∗^*P* < 0.05.

**Figure 2:**
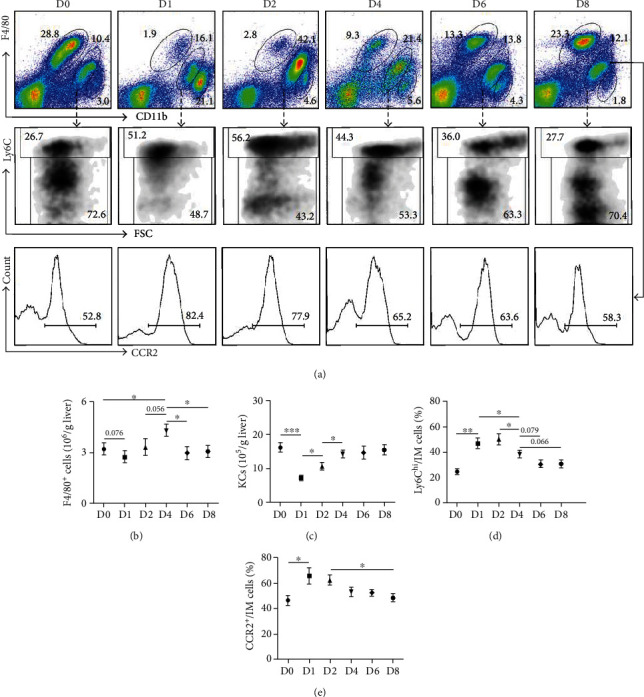
The reduction and restoration of Kupffer cells and the transformation of ly6C^hi^ infiltrated macrophages after PHx. (a) C57BL/6 mice were sacrificed at day 1, 2, 4, 6, or 8 after 2/3 PHx or sacrificed immediately after sham operation. The subpopulation of HNPCs was analyzed by FACS with anti-F4/80, anti-CD11b, anti-ly6C, and anti-CCR2 antibodies. The number of (b) F4/80^+^ macrophages and (c) F4/80^hi^ CD11b^+^ KCs in (a) was determined and quantitatively compared. The percentage of (d) ly6C^hi^ IMs (D) and (e) CCR2^+^ IMs in (a) was determined and quantitatively compared. Bars = means ± SD, *n* = 8. ^∗^*P* < 0.05, ^∗∗^*P* < 0.01, and ^∗∗∗^*P* < 0.001.

Hepatic proliferation was examined by IHC staining with anti-Ki67 antibody at different time points after PHx. The number of Ki67^+^ cells peaked at day 2 (D2) and reverted to baseline (D0) at D8 (Figures 1(a) and 1(b)). According to the morphological observation, most of them were hyperplastic hepatocytes at D2 or proliferative hepatic nonparenchymal cells (HNPCs) at D4. This was consistent with previous studies that hepatocytes and HNPCs reached the proliferation peak at D2 and D4 after PHx, respectively[13]. The number of macrophages was counted by IHC staining and FACS assay using anti-F4/80 antibody, and the results showed that the number of hepatic macrophages reduced slightly at D1, but peaked at D4, and then gradually returned to the baseline at D8 after PHx (Figures 1(c) and 2(b)). Moreover, CD68, one classical marker of KCs [3], was analyzed using IHC; the result showed that the number of CD68^+^ KCs markedly declined at D1 after PHx (Figures 1(a) and 1(d)).

Based on the expression level of CD11b and F4/80 on macrophages, the hepatic macrophages could be divided into two populations [5, 13], one is F4/80^hi^CD11b^+^ KCs and another is F4/80^+^CD11b^hi^ IMs (Supplementary Figure 1A). Both the percentage and the cell number of F4/80^hi^CD11b^+^ KCs dramatically reduced at the early time after PHx and then gradually restored (Figures 2(a) and 2(c)); this trend was consistent with the dynamic change of CD68^+^ KCs in Figure 1. These results suggested that the reduction of hepatic macrophages might be attributed to the reduction of KCs at the early stage after PHx. Meanwhile, the percentage of F4/80^+^CD11b^hi^ IMs increased at D1 and peaked at D2 and then gradually reverted to baseline at D8 (Figure 2(a)). Recruitment of IMs was potentially related to the augment of the hepatic macrophage pool. These results indicated that recruitment of IMs into regenerative liver might also contribute to the increased number of hepatic macrophages at D4 after PHx. Taken together, these results demonstrated that the dynamic change of hepatic macrophages during liver regeneration might relate with their origin after PHx.

### 3.2. Dynamic Change of Different Functional Subsets of Hepatic Macrophages after PHx

The hepatic IMs were heterogeneous with the distinct expression level of ly6C and CCR2 (Supplementary Figure 1B). After PHx, the percentage of ly6C^hi^ IMs or CCR2^+^ IMs both increased significantly at the early stage (D1 and D2) and then gradually descended to the baseline at D8 (Figures 2(a), 2(d), and 2(e)). In addition, CD11b^+^ly6C^hi^ monocytes also increased significantly in bone marrow (BM) and peripheral blood (PB) earlier after PHx (Supplementary Figure 2). This result meant that many ly6C^hi^CCR2^+^ monocytes were agitated and recruited to the liver earlier after PHx. During liver regeneration, the ly6C^hi^CCR2^+^ monocytes differentiated into ly6C^hi^ IMs that gradually adopted the phenotypic switch to ly6C^lo^ IMs.

**Figure 3:**
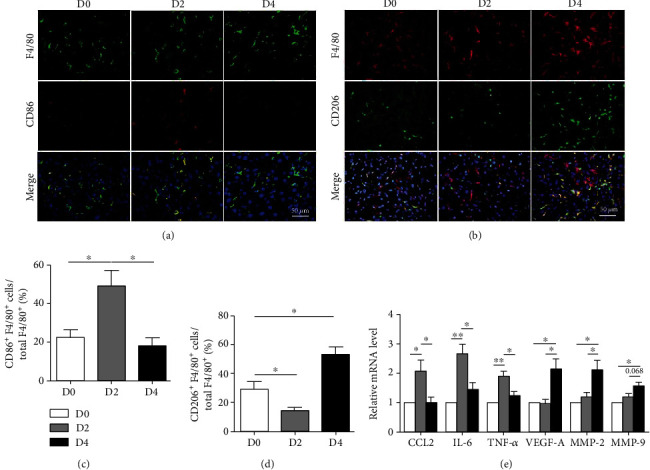
The functions of activated macrophages were determined after PHx. (a, b) C57BL/6 mice were sacrificed at D2 or D4 after 2/3 PHx or sacrificed immediately after sham operation. Liver sections were stained with (a) anti-F4/80 and anti-CD86 antibodies or (b) anti-F4/80 and anti-CD206 immunofluorescence and counterstained with Hoechst. (c, d) The percentage of double-positive cells in macrophages were determined and quantitatively compared in (a, b). (e) F4/80^+^ macrophages were sorted by MACS from HNPCs, and the mRNA levels of CCL2, IL-6, TNF-*α*, VEGF-A, MMP-2, and MMP-9 were determined by qRT-PCR. Bars = means ± SD, *n* = 5. ^∗^*P* < 0.05, ^∗∗^*P* < 0.01.

The previous studies showed that the ratio of ly6C^hi^ to ly6C^lo^ macrophage represented the functional states of IMs in liver injury [4, 5, 12]. The switch of ly6C^hi^ to ly6C^lo^ subset suggested that hepatic macrophage might exert different function at the different phases after PHx. To further elucidate this question, the expression profile of hepatic macrophages was detected at D2 (the peak of hepatocyte proliferation) and D4 (the peak of HNPC proliferation). Meanwhile, the percentage of ly6C^hi^ and ly6C^lo^ IMs was significantly different between these two time points. As we know, the CD86 and CD206 were representative markers of M1 (classical) and M2 (alternative) macrophages, respectively. Using immunofluorescence staining, we found that the hepatic macrophages showed more CD86 expression and less CD206 expression at D2 after PHx, while the phenomena were totally reversed at D4 (Figures 3(a)–3(d)). Then, the hepatic macrophages were isolated by MACS and the expression of related molecules during liver regeneration was detected by qRT-PCR. The levels of CCL2, IL-6, and TNF-*α* were significantly upregulated in the macrophages at D2, while those of MMP-2, MMP-9, and VEGF-A were significantly increased in the macrophages at D4 after PHx (Figure 3(e)). These results suggested that different haptic macrophage subsets indeed possessed different functions during liver regeneration after PHx. Namely, in the early inductive phase, hepatic macrophages, presenting the M1-like phenotype with the higher level of IL-6 and TNF-*α* expression, were strongly related with hepatocyte proliferation; in the subsequent angiogenic phase, hepatic macrophages, presenting M2-like phenotype with the high level of VEGF-A and MMP expression, might participate in the regulation of angiogenesis. Collectively, these results indicated that liver regeneration could depend on different function subsets of hepatic macrophages after PHx.

### 3.3. The Deletion of CCR2 Delayed Hepatocyte Proliferation and Subsequent Liver Regeneration after PHx in Mice

**Figure 4:**
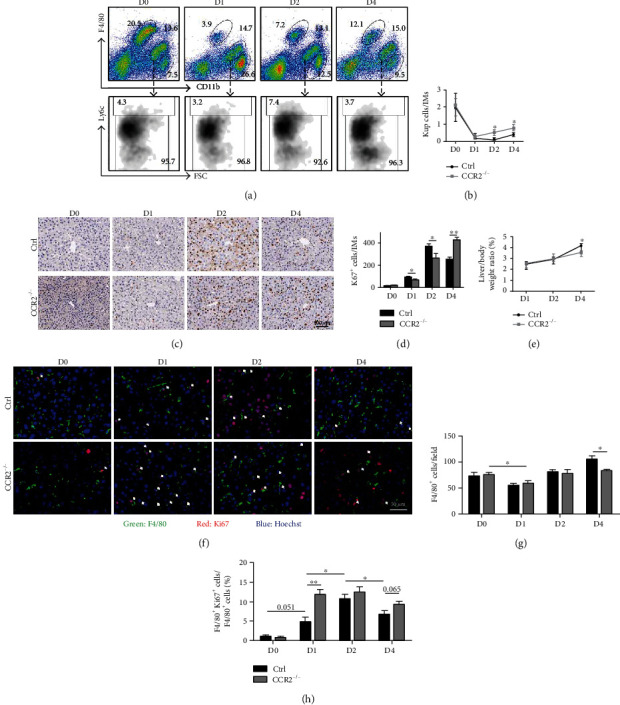
Liver regeneration was delayed when infiltration of circulating monocyte was blocked in CCR2^−/−^ mice after PHx. (a) C57BL/6 mice and CCR2-KO mice were sacrificed D1, 2, or 4 after 2/3 PHx or sacrificed immediately after sham operation. The subpopulation of HNPCs in control and CCR2-KO mice was analyzed by FACS with anti-F4/80, anti-CD11b, and anti-ly6C antibodies at the indicated times after PHx. (b) The ratios of KCs/IMs in (a) were determined and quantitatively compared. (c) Liver sections were stained with anti-Ki67 IHC. (d) The number of Ki67^+^ cells in (c) was counted and quantitatively compared. (e) Liver-body weight ratios of control and CCR2-KO mice were quantitatively compared at the indicated times. (f) Liver sections stained with anti-F4/80 and anti-Ki67 immunofluorescence and counter-stained with Hoechst. The arrow indicated the double-positive cells. (g) The number of F4/80^+^ cells in (f) was counted and quantitatively compared. (h) The percentage of double-positive cells in macrophages was determined and quantitatively compared in (f). Bars = means ± SD, *n* = 4. ^∗^*P* < 0.05, ^∗∗^*P* < 0.01.

To verify the role of hepatic IMs in liver regeneration, we further used CCR2-KO mice with the PHx model. Compared to the control mice, the CD11b^+^ly6C^hi^ BM monocytes were accumulated more in BM of CCR2^−/−^ mice after PHx (Supplementary Figure 2A). As expected, in the PB of CCR2^−/−^ mice, almost no CD11b^+^ly6C^hi^ monocytes appeared after PHx (Supplementary Figure 2B). Consequently, in CCR2^−/−^ mice, the percentage of hepatic IMs showed no obvious increase as that in control mice after PHx, meanwhile ly6C^hi^ IMs existed rarely (Figure 4(a)). The ratio of KCs to IMs was ascended since D2 in CCR2^−/−^ mice compared to that in control mice after PHx (Figure 4(b)). Therefore, these results indicated that the ly6C^hi^ monocytes could not migrate into the PB and liver tissue on the background of CCR2 deficiency, as well as they could not participate in liver regeneration after PHx.

Next, the effect of CCR2 knockout on liver regeneration was further investigated using IHC. Compared with control mice, the peak of hepatocyte proliferation appeared at D4 and was delayed two days in CCR2^−/−^ mice (Figures 4(c) and 4(d)); consequently, the ratio of liver to body weight was also decreased at D4 in CCR2^−/−^ mice (Figure 4(e)), suggesting that the ability of liver regeneration was retarded in CCR2-deficient mice. In summary, these results indicated that CCR2^+^ly6C^hi^ IMs were necessary macrophage subsets for liver regeneration after PHx.

### 3.4. The Restoration of Hepatic Macrophages Both Depended on Self-Renew of KCs and Replenishment of IMs

Furthermore, we investigated whether the blockade of circulating monocyte infiltration could affect hepatic macrophage restoration after PHx. Although the number of hepatic F4/80^+^ cells showed no difference between CCR2^−/−^ and control mice at D0, D1, and D2 after PHx, their numbers reduced significantly at D4 in CCR2^−/−^ mice compared to the control mice (Figures 4(f) and 4(g)). This result indicated that replenishment of ly6C^hi^ IMs was necessary for restoration of hepatic macrophages after PHx. After that, we further detected macrophage proliferation. Consistent with the previous study [14], the macrophage proliferation peaked at D2 after PHx in control mice; however, in CCR2^−/−^ mice, the peak of macrophage proliferation occurred at D1 after PHx and was maintained longer (Figures 4(f) and 4(h)). KCs, ly6C^hi^ IMs, and ly6C^lo^ IMs could own the capacity of proliferation after liver injury, and the ly6C^hi^ IMs even were the largest subsets of proliferative macrophages in all of the time points during liver inflammation [5, 15]. However, given the absence of ly6C^hi^ IMs and the increasing proportion of KCs (Figures 4(a) and 4(b)) in CCR2^−/−^ mice, the acceleration of macrophage proliferation might be mainly attributed to KC proliferation. Nevertheless, the enhancement of KC proliferation did not compensate completely for the loss of CCR2^+^ly6C^hi^ IMs in the liver of CCR2^−/−^ mice. Collectively, these results suggested that the restoration of hepatic macrophages could attribute to the self-renew of KCs and replenishment of IMs after PHx.

## 4. Discussion

Hepatic macrophages are heterogeneous in origin and phenotype according to local tissue signals and disease progression, which play a critical role in disease progression. In the present study, we focused on the dynamics of diverse origin and different functional macrophages during liver regeneration utilizing the 2/3 PHx mouse model.

In this study, our results demonstrated that KCs dramatically reduced at D1 after PHx. KCs act as “gatekeepers,” determining the initiation or suppression of the immune response. During homeostasis, KCs maintain a tolerogenic environment dependent on highly effective phagocytic and scavenging mechanisms [2]. When liver tissue encounters with various injuries, such as toxin [4] or infection [16], KCs are activated by kinds of factors to form the inflammasome, which not only promote the release of potent proinflammatory signaling molecules but also mediate the apoptosis [17] or pyroptosis [18] of KCs. These reactions ultimately lead to the reduction of KCs and the recruitment of circulating monocytes and the dramatical changes of the hepatic macrophage pool. In our study, we found that KCs also presented dramatically reduction at the early stage during liver regeneration after PHx. Lipopolysaccharide (LPS) maybe a reasonable candidate to promote KC activation and induce KC death after PHx, because it increases significantly in the remnant liver under augmented portal vein pressure and blood flow after PHx [19]. Elevating LPS promotes the switch of KCs from immunosuppression to immunoactivation and triggers liver regeneration [19]. Therefore, gut-derived endotoxin elicits hepatic nutritional factors and liver regeneration is depressed in germ-free and LPS-resistant mice after PHx [20].

It is debatable whether circulating monocytes are recruited and maintain murine hepatic macrophage pools after PHx [14, 21]. These controversies indicate the importance and complexity of macrophages in liver regeneration. Here, our results showed that the number of hepatic macrophages reduced slightly at D1, peaked at D4, and gradually reached the baseline at D8 and the percentage of F4/80^+^CD11b^hi^ IMs increased between D1 and D4 and peaked at D2 after PHx. Meanwhile, the percentage of ly6C^hi^ and CCR2^+^ IMs increased significantly at the early time after PHx. In addition, in CCR2^−/−^ mice, the number of hepatic F4/80^+^ cells significantly reduced at D4 after PHx and the peak of hepatocyte proliferation was delayed. All of these results suggested that the restoration of hepatic macrophages and liver regeneration both depended on infiltration and replenishment of circulating monocyte-derived macrophages after PHx.

In kinds of liver injury models [4, 5], ly6C^hi^ IMs derived from CCR2^+^ly6C^hi^ monocytes play proinflammatory and profibrosis roles and they subsequently mature into ly6C^lo^ IMs in situ, which present prorestorative phenotype and promote inflammation and fibrosis resolution. The ratio of ly6C^hi^ to ly6C^lo^ subsets represented the functional states of IMs in liver injury. In our study, the percentage of ly6C^hi^ IMs increased significantly at the early time after PHx, and then, it gradually decreased and returned to the baseline. Meanwhile, the expression profile of hepatic macrophages was significantly different at D2 and D4 after PHx. At D2, hepatocyte proliferation reached the peak and hepatic macrophages presented an M1-like phenotype with high levels of CCL2, IL-6 and TNF-*α* expression; at D4, HNPC proliferation reached the peak and hepatic macrophages exhibited M2-like phenotype with a high level of MMP-2, MMP-9, and VEGF-A expression. The expression profile of ly6C^hi^ or ly6C^lo^ macrophages after PHx in our study was consistent with the expression pattern in the liver injury model of previous studies [4, 5]. It is very interesting that the dynamics of the hepatic macrophage phenotype during liver regeneration is analogous to that in liver sterile injury. In the early inductive phase, a large number of ly6C^hi^ IMs presented a proinflammatory phenotype and secreted cytokines, such as IL-6 and TNF-*α*, which initiated progression of hepatocyte proliferation. Meanwhile, the high level of CCL2 expression promoted the recruitment of the circulating monocyte. Accompanied with termination of hepatocyte proliferation, ly6C^hi^ IMs subsequently matured into ly6C^lo^ IMs that could participate in the proliferation of endothelial cells and the reconstitution of regenerative liver mass. The VEGF-A-VEGFR2 axis mediating proliferative angiogenesis is required for liver regeneration [13]. Gelatinase, MMP-2, and MMP-9 play an important role in liver regeneration after PHx. At very early stage after PHx, these molecules are elevated and activated and then participate in the matrix degradation which may cause rapid release and accumulation of HGF [22]. At the late stage, followed by angiogenesis, continuous capillaries are converted into sinusoids and MMPs may participate in remodeling process [23]. Therefore, in the late angiogenic phase, ly6C^lo^ IMs were strongly related with angiogenesis and reconstitution.

Following liver injury or massive KC depletion, IMs can reconstitute the liver-resident macrophage pool. Namely, IMs can replenish the hepatic macrophage population and transform to KCs. Interestingly, Guilliams and Scott brought forth the niche model to explain the origin of tissue-resident macrophage under inflammation [24]. The niche model could also support our findings in which the hepatic macrophage pool was restored after PHx. Firstly, hepatic macrophage niche was accessible because there was no barrier for precursor to entrance into the liver after PHx; secondly, the niche was available because the number of KCs declined significantly at D1 after PHx; finally, there were two competing cells present after PHx; one was ly6C^hi^ IMs that infiltrated into the liver as well as harbored fascinating plasticity according to the tissue microenvironment and another were remnant KCs that had the ability to self-renew in situ. Therefore, after PHx, hepatic macrophage niche could permit the replenishment of IMs and KC self-renewal; both of them contributed to liver regeneration.

## 5. Conclusion

It is traditionally considered that liver regeneration is not related to the tissue damage and inflammation. But our results suggest that liver regeneration, like most of liver sterile injury and infection, is finely regulated by different ontogenic and functional macrophage subsets. There is the classically dynamic change of liver macrophages while there are liver regeneration after PHx: after liver resection, hepatic-resident macrophages, KCs, are activated and then reduced greatly; chemokines initiate the ly6C^hi^ monocytes emigrating from bone marrow into the liver; in the inductive phase, ly6C^hi^ IMs, presenting a proinflammatory phenotype, express high levels of IL6 and TNF-*α*, which are indispensable cytokines for hepatocyte proliferation; in the subsequent angiogenic phase, ly6C^hi^ IMs gradually mature into ly6C^lo^ subsets; ly6C^lo^ IMs, expressing a high level of VEGF-A and MMPs, participate in proliferation of vascular endothelial and reconstruction of liver mass; finally, ly6C^lo^ IMs may transform toward fully functioning KCs and become IM-derived KCs that restore the hepatic macrophage pool with locally proliferating KCs together. All these results suggest that targeting macrophage therapy should consider their heterogeneity during liver regeneration.

## Data Availability

The data can be obtained from the corresponding author with consent.

## Supplementary Materials

Supplementary Materials Supplementary Figure 1: gating strategy of hepatic myeloid cells. (A) The hepatic nonparenchymal cells were isolated from C57BL/6 mice and analyzed by FACS. Circles in c. indicated the liver-resident macrophages (KCs, F4/80^hi^CD11b^+^), the infiltrating monocytes-derived inflammatory macrophages (IMs, F4/80^+^CD11b^hi^), and neutrophils (Ns, F4/80^−^CD11b^hi^). (B) The expression of ly6C or CCR2 of the different myeloid cells in (A) was analyzed by FACS. Supplementary Figure 2: the bone marrow myeloid cells were both augmented in CCR2-KO and WT mice after PHx, but these cells could not emigrate into the peripheral blood and liver in CCR2-KO mice. (A) C57BL/6 mice and CCR2-KO mice were sacrificed D1, 2, or 4 after 2/3 PHx or sacrificed immediately after sham operation. The subpopulation of bone marrow myeloid cells was analyzed by FACS. (B) The subpopulation of peripheral blood myeloid cells was analyzed by FACS. *n* = 4. Supplementary Table 1: antibodies used in this study. Supplementary Table 2: primers used for RT-PCR in this study.
