# Supplementary material for: The Contribution of Hepatic Macrophage Heterogeneity during Liver Regeneration after Partial Hepatectomy in Mice
Source: J Immunol Res. 2022 Oct 7;2022:3353250. doi: 10.1155/2022/3353250 (PMC9568332; doi:10.1155/2022/3353250)
Supplement: Supplementary Materials — Supplementary Figure 1: gating strategy of hepatic myeloid cells. (A) The hepatic nonparenchymal cells were isolated from C57BL/6 mice and analyzed by FACS. Circles in c. indicated the liver-resident macrophages (KCs, F4/80hiCD11b+), the infiltrating monocytes-derived inflammatory macrophages (IMs, F4/80+CD11bhi), and neutrophils (Ns, F4/80−CD11bhi). (B) The expression of ly6C or CCR2 of the different myeloid cells in (A) was analyzed by FACS. Supplementary Figure 2: the bone marrow myeloid cells were both augmented in CCR2-KO and WT mice after PHx, but these cells could not emigrate into the peripheral blood and liver in CCR2-KO mice. (A) C57BL/6 mice and CCR2-KO mice were sacrificed D1, 2, or 4 after 2/3 PHx or sacrificed immediately after sham operation. The subpopulation of bone marrow myeloid cells was analyzed by FACS. (B) The subpopulation of peripheral blood myeloid cells was analyzed by FACS. n = 4. Supplementary Table 1: antibodies used in this study. Supplementary Table 2: primers used for RT-PCR in this study. [file 3353250.f1.zip › Supplementary data (2).docx]

**Supplementary data**


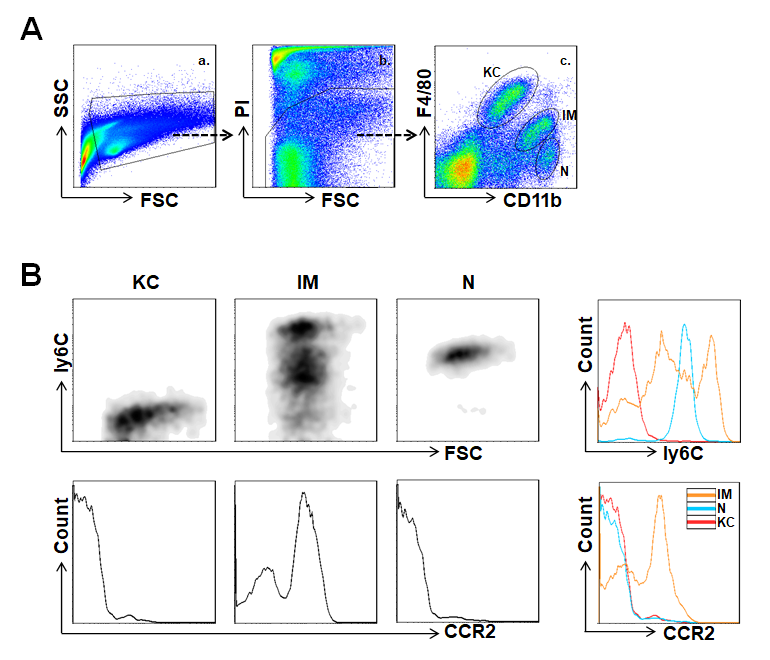


**Supplementary Figure 1. Gating strategy of hepatic myeloid cells.**

Note: (A) The hepatic non-parenchymal cells were isolated from C57BL/6 mice, and analyzed by FACS. Circles in c. indicated the liver resident macrophages (KCs, F4/80^hi^CD11b^+^), the infiltrating monocytes-derived inflammatory macrophages (IMs, F4/80^+^CD11b^hi^), and neutrophils (Ns, F4/80^-^CD11b^hi^). (B) The expression of ly6C or CCR2 of the different myeloid cells in (A) was analyzed by FACS.


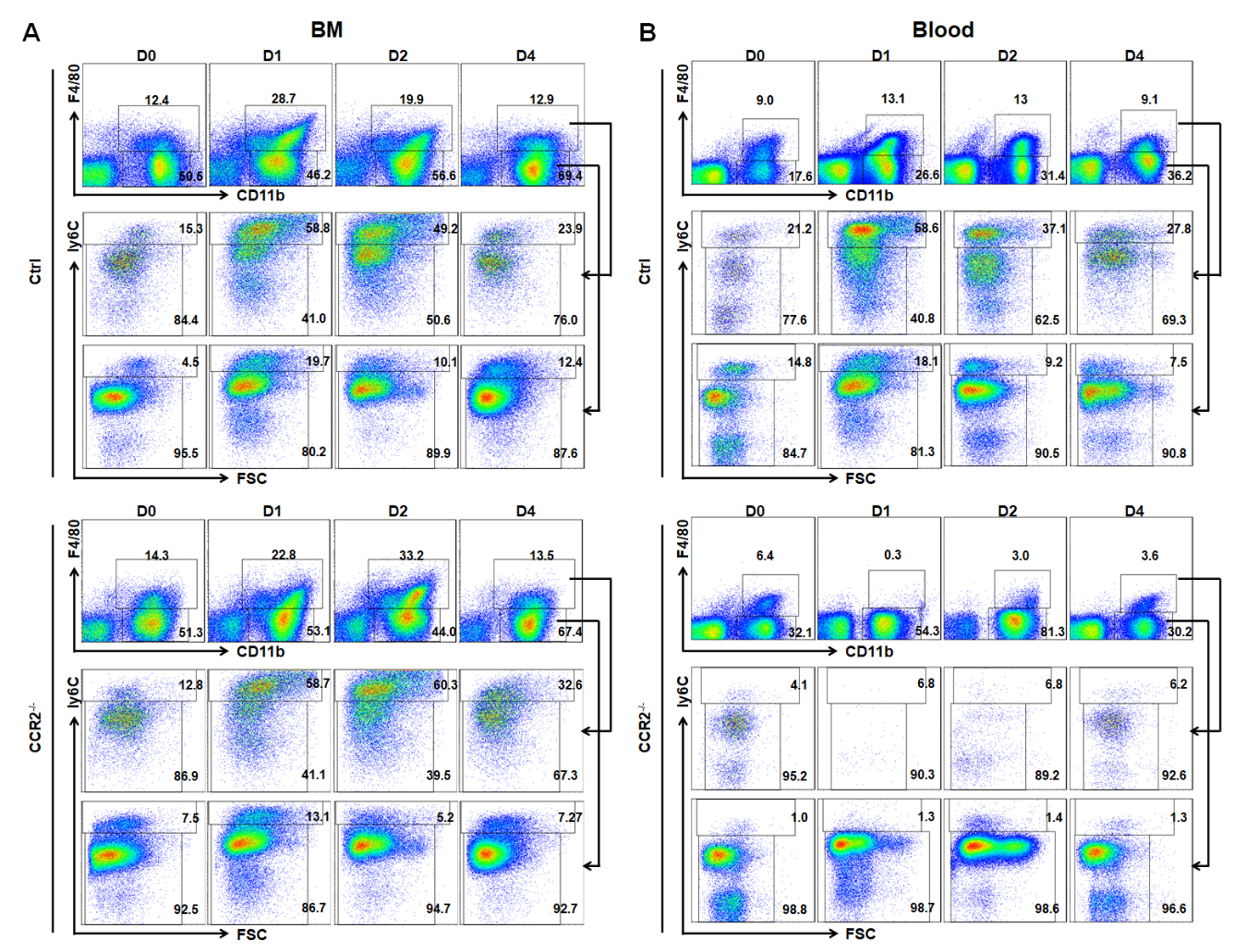


**Supplementary Figure 2. The bone marrow myeloid cells were both augmented in CCR2-KO and WT mice after PHx, but these cells could not emigrate into the** **peripheral blood and liver in CCR2-KO mice.**

Note: (A) C57BL/6 mice and CCR2-KO mice were sacrificed D1, 2 or 4 after 2/3 PHx, or sacrificed immediately after [sham](javascript:;) [operation](javascript:;). The subpopulation of bone marrow myeloid cells was analyzed by FACS. (B) The subpopulation of peripheral blood myeloid cells was analyzed by FACS. n = 4.

**Supplementary Table 1. Antibodies used in this study.**

| **Name** | **Supplier** | **Clone #** | **Purpose** |
| --- | --- | --- | --- |
| Alexa 488 F4/80  APC CD11b  PE CD11b  APC Ly6C  PE CCR2  PE CD45  rat anti-F4/80  rabbit anti-Ki67  rabbit anti-CD68  rabbit anti-CD86  rabbit anti-CD206  Alexa 594 anti-rat IgG  Alexa 488 anti-rat IgG  Cy3 anti-rabbit IgG  Alexa 488 anti-rabbit IgG  HRP goat anti-rabbit IgG  Biotin anti-rat IgG  Streptavidin-peroxidase | Biolegend  Biolegend  BD Pharmingen  Biolegend  R&D Systems  eBioscience  eBioscience  Lab Vision  Servicebio  Servicebio  Servicebio  Life Technologies  Life Technologies  Sigma  Servicebio  Boster Bio Tec  Boster Bio Tec  Boster Bio Tec | BM8  M1/70  M1/70  HK1.4  475301  30-F11  BM8  SP6 | FACS  FACS  FACS  FACS  FACS  FACS  IF, IHC  IF, IHC  IHC  IF  IF  IF  IF  IF  IF  IHC  IHC  IHC |

Supplementary Table 2. Primers used for RT-PCR in this study.

| **Name** | **Sequence** |
| --- | --- |
| TNF-α F  R  IL-6 F  R  CCL2 F  R  MMP-2 F  R  MMP-9 F  R  VEGF-A F  R  β-actin F  R | 5'-CAGGAGGGAGAACAGAAACTCCA  5'-CCTGGTTGGCTGCTTGCTT  5'-CCACTTCACAAGTCGGAGGCTTA  5'-GCAAGTGCATCATCGTTGTTCATAC  5'-TTAAAAACCTGGATCGGAACCAA  5'-GCATTAGCTTCAGATTTACGGGT  5'-GATAACCTGGATGCCGTCGTG  5'-CTTCACGCTCTTGAGACTTTGGTTC  5'-CCATGCACTGGGCTTAGATCA  5'-GGCCTTGGGTCAGGCTTAGA  5'-GCACATAGAGAGAATGAGCTTCC  5'-CTCCGCTCTGAACAAGGCT  5'-CATCCGTAAAGACCTCTATGCCAAC  5'-ATGGAGCCACCGATCCACA |
