# Supplementary figures and images for: The Contribution of Hepatic Macrophage Heterogeneity during Liver Regeneration after Partial Hepatectomy in Mice
Source: J Immunol Res. 2022 Oct 7;2022:3353250. doi: 10.1155/2022/3353250 (PMC9568332; doi:10.1155/2022/3353250)

**A**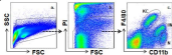**B**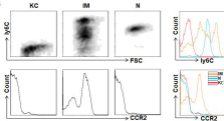

Supplement: Supplementary Materials — Supplementary Figure 1: gating strategy of hepatic myeloid cells. (A) The hepatic nonparenchymal cells were isolated from C57BL/6 mice and analyzed by FACS. Circles in c. indicated the liver-resident macrophages (KCs, F4/80hiCD11b+), the infiltrating monocytes-derived inflammatory macrophages (IMs, F4/80+CD11bhi), and neutrophils (Ns, F4/80−CD11bhi). (B) The expression of ly6C or CCR2 of the different myeloid cells in (A) was analyzed by FACS. Supplementary Figure 2: the bone marrow myeloid cells were both augmented in CCR2-KO and WT mice after PHx, but these cells could not emigrate into the peripheral blood and liver in CCR2-KO mice. (A) C57BL/6 mice and CCR2-KO mice were sacrificed D1, 2, or 4 after 2/3 PHx or sacrificed immediately after sham operation. The subpopulation of bone marrow myeloid cells was analyzed by FACS. (B) The subpopulation of peripheral blood myeloid cells was analyzed by FACS. n = 4. Supplementary Table 1: antibodies used in this study. Supplementary Table 2: primers used for RT-PCR in this study. [file 3353250.f1.zip › Supplementary Figure1.pdf]

**A****BM**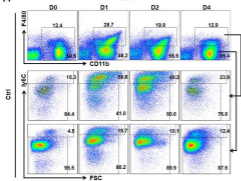**B****Blood**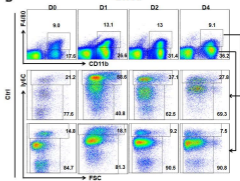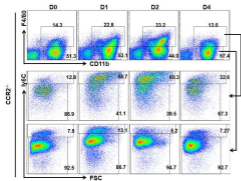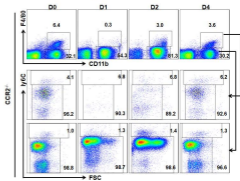

Supplement: Supplementary Materials — Supplementary Figure 1: gating strategy of hepatic myeloid cells. (A) The hepatic nonparenchymal cells were isolated from C57BL/6 mice and analyzed by FACS. Circles in c. indicated the liver-resident macrophages (KCs, F4/80hiCD11b+), the infiltrating monocytes-derived inflammatory macrophages (IMs, F4/80+CD11bhi), and neutrophils (Ns, F4/80−CD11bhi). (B) The expression of ly6C or CCR2 of the different myeloid cells in (A) was analyzed by FACS. Supplementary Figure 2: the bone marrow myeloid cells were both augmented in CCR2-KO and WT mice after PHx, but these cells could not emigrate into the peripheral blood and liver in CCR2-KO mice. (A) C57BL/6 mice and CCR2-KO mice were sacrificed D1, 2, or 4 after 2/3 PHx or sacrificed immediately after sham operation. The subpopulation of bone marrow myeloid cells was analyzed by FACS. (B) The subpopulation of peripheral blood myeloid cells was analyzed by FACS. n = 4. Supplementary Table 1: antibodies used in this study. Supplementary Table 2: primers used for RT-PCR in this study. [file 3353250.f1.zip › Supplementary Figure2.pdf]
